# *Amblyomma aureolatum* Genetic Diversity and Population Dynamics Are Not Related to Spotted Fever Epidemiological Scenarios in Brazil

**DOI:** 10.3390/pathogens10091146

**Published:** 2021-09-06

**Authors:** Karla Bitencourth, Marinete Amorim, Stefan Vilges de Oliveira, Gilberto Salles Gazêta

**Affiliations:** 1 Laboratório de Referência Nacional em Vetores das Riquetsioses, Fundação Oswaldo Cruz, Rio de Janeiro 21045-900, RJ, Brazil; mamorim@ioc.fiocruz.br (M.A.); gsgazeta@ioc.fiocruz.br (G.S.G.); 2 Programa de Pós-Graduação em Biodiversidade e Saúde, Fundação Oswaldo Cruz, Rio de Janeiro 21045-900, RJ, Brazil; 3 Secretaria de Vigilância em Saúde, Ministério da Saúde, Unidade Técnica de Vigilância de Zoonoses, Brasília 70719-040, DF, Brazil; stefanbio@yahoo.com.br; 4 Faculdade de Medicina, Universidade Federal de Uberlândia, Uberlândia 38400-902, MG, Brazil

**Keywords:** “*ovale* complex”, Atlantic Forest, phylogeography, population genetics, population growth, rickettsiosis, yellow dog tick

## Abstract

Regional differences in tick-borne disease epidemiology may be related to biological variations between vector populations. *Amblyomma aureolatum* (Ixodida: Ixodidae), a neotropical tick, is known from several regions in Brazil. However, only in the metropolitan area of São Paulo (SP) state are there studies that establish its role as a vector of a pathogenic rickettsia (*Rickettsia rickettsii*). The aim of the study was to analyze the genetic diversity, population dynamics, and rickettsia infection in *A. aureolatum* populations from different spotted fever scenarios in Brazil. Samples were subjected to DNA extraction, amplification and sequencing of 12S rDNA, cytochrome oxidase subunit II and D-loop mitochondrial markers for tick population analyses, and *glt*A, *htr*A, *omp*A, and *omp*B genes for rickettsia researches. Of the 7–17 tick haplotypes identified, 5–13 were exclusive to each population and 2–12 for each epidemiological scenario, as well as three haplotypes shared by all populations. *Amblyomma aureolatum* populations are expanding, and do not appear to be genetically structured vis-a-vis the different epidemiological scenarios studied. *Rickettsia bellii* (in SP) and *Rickettsia felis* (in Santa Catarina) were identified as infecting *A. aureolatum*. No relationship between tick haplotypes and rickettsia types were observed.

## 1. Introduction

Ticks are very important to world public health as vectors of a variety of bioagents. Brazilian spotted fever (BSF), the main tick-borne zoonotic disease in Brazil, is caused by *Rickettsia rickettsii* and transmitted by *Amblyomma sculptum* Berlese, 1888 (the star tick) and *Amblyomma aureolatum* (the yellow dog tick) [1]. Knowledge of BSF vectors and epidemiology, including the vector–pathogen–host interaction, is key for effective disease control strategies in the context of One Health. However, such knowledge is still incipient, especially for *A. aureolatum*.

Records of *A. aureolatum* are restricted to the neotropical region, being found in the southeastern region of South America, from Uruguay to Surinam, including records for northeastern Argentina, eastern Paraguay, French Guiana, and Brazil (largely in the states of the south and southeast regions) [2]. Atlantic Forest, with conditions of humidity and high temperature throughout the year, appears to be the natural habitat of this ixodid [3]. However, there are records of *A. aureolatum* in the Pampa biome, in southern Brazil, where winters reach freezing, but summers are hot and dry e.g., [4,5,6].

Immature stages of *A. aureolatum* feed on passerine birds and a few rodent species [2,7]. In forest areas, the main hosts for both sexes of this tick are members of the order carnivora, among them the crab-eating fox (*Cerdocyon thous*), and the crab-eating raccoon (*Procyon cancrivorus*). Thus, BSF cases associated with *A. aureolatum* seem to occur where human dwellings are close to forest fragments and domestic dogs have free access to it. Dogs get infested by adult *A. aureolatum*, then go on to carry this ixodid into their owners’ households [1]. Humans are parasitized only by adult ticks, usually by a single specimen [5], as the population density of this species is usually low [3].

Although populations of *A. aureolatum* are present, under similar environmental conditions (human habitations close to forest fragments and dogs infested with *A. aureolatum*), in several locations in Brazil, information concerning the distribution of BSF cases associated with this ixodid are restricted to the metropolitan area of São Paulo (SP) state (southeast region) [1], where the endemic sites for the disease differ from non-endemic sites due to the presence of smaller and more degraded areas of the Atlantic Forest, with low vertebrate abundance and richness [7,8]. Despite this and the fact that the highest known extent of *A. aureolatum R. rickettsii* vectoring occurs within the SP metropolitan region, this BSF scenario may have a wider geographic distribution, one which is currently unreported solely due to the scarcity of studies in other regions of Brazil.

Regional differences in the epidemiology of tick-borne diseases (TBD), such as BSF, may be related to pathogen behavior and biological variations between vector populations [9]. Intraspecific genetic studies of ticks can provide information on their variability, dispersal mechanisms, gene flow, population structure, and expansion, as well as TBD epidemiology and surveillance [10,11,12,13]. However, this knowledge is scarce for *A. aureolatum*, with just one study for SP, Brazil [14]. Accordingly, the current study aimed to analyze the genetic diversity, population dynamics, and infection by rickettsia of *A. aureolatum* populations from different spotted fever (SF) scenarios in Brazil.

## 2. Results

### 2.1. Molecular Identification of Ticks

Following molecular tests, 90 sequences of 12S rDNA (371 bp), 58 sequences of cytochrome oxidase subunit II (COII) (487 bp), and 110 sequences of D-loop (458 bp) were obtained. Of these markers, 12S rDNA was the only one with sequences available in GenBank for *A. aureolatum*, and with these the sequences of the current study gave similar values (99.42–100.00% with sequences from Paraná State, Brazil: AY277248-49).

### 2.2. Intrapopulational Analysis: A. aureolatum Has Unique Haplotypes per Population and Low Nucleotide Diversity

Based on the genetic variation of 12S rDNA, COII, and D-loop sequences, 7 (hI–hVII), 17 (h1–h17), and 14 (hA–hN) *A. aureolatum* haplotypes were identified, respectively (Tables S1–S3).

In the 371–bp data matrix of 12S rDNA, the Rio Grande do Sul (RS) population showed the highest indices for polymorphic site (5), haplotype (0.787), and nucleotide diversity (0.0046) (Table 1). Of the seven haplotypes identified, hI (51.12%) and hII (40.00%) were the most frequent, with the latter shared by all populations. Unique haplotypes were found in RS (hIII–hV) and Paraná (PR) (hVI and hVII) populations (Table S1).

Analysis of 487 nucleotides of the *A. aureolatum* COII gene identified two (Rio de Janeiro (RJ)) to 10 (RS) polymorphic sites that defined the 17 haplotypes described in *A. aureolatum* populations studied in Brazil (Table 1 and Table S2). Of these, the most frequent was h1 (31.04%), found in PR and Santa Catarina (SC), while h15 was shared by all populations (27.60%). Other haplotypes shared between populations were h2 (SC and São Paulo (SP)), h4 (PR and SC), and h14 (PR, RS, and SP). All populations had unique, low-frequency haplotypes: PR (h11–h13), SC (h3), RJ (h5), RS (h8–h10, h16, and h17), and SP (h6 and h7) (Table S2), where wide haplotype diversity (0.911–0.533) and low nucleotide diversity (0.0048–0.0015) were observed (Table 1).

Of the 14 *A. aureolatum* D-loop haplotypes, hA was the most frequent (70.01%) and identified in all populations. Other haplotypes shared among populations were hB (PR, SC, RJ, and SP), hC (PR and SC), and hF (PR and RJ). Unique haplotypes were found in all populations: hD (SC), hE (RJ), hG–hJ (SP), hK–hL (RS), and hM–hN (PR); with the exception of hK, all of the other haplotypes showed low frequency (Table S3). The 458 bp fragments aligned from D-loop revealed three (SC and RJ) to six (PR and SP) polymorphic sites, resulting in the low nucleotide (0.0007 in SC; 0.0042 in RS) and haplotype diversity (0.331 in SC; 0.601 in PR) detected in *A. aureolatum* populations (Table 1).

For all of the analyzed markers, in general, Group I populations (PR, SC, RJ, and RS) had higher diversity index values and more haplotypes than the population from Group II (SP) (Table 1 and Tables S1–S3).

**Figure 1:**
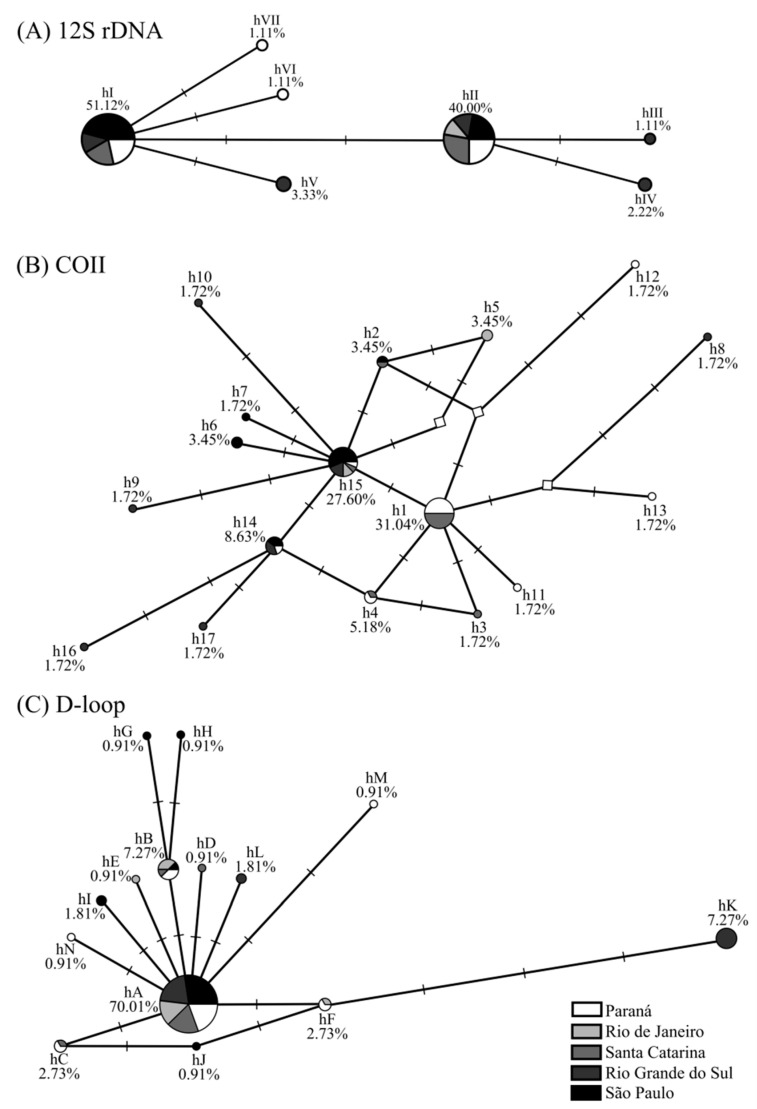
(**A**) Haplotype network for 12S rDNA, (**B**) cytochrome oxidase subunit II (COII), and (**C**) D-loop markers from *Amblyomma aureolatum* collected under different spotted fever scenarios in Brazil and their relative frequencies. Networks were generated with the median-joining method, using maximum parsimony post-processing. The circle size corresponds to the frequency of each haplotype sequence, each dash on the line (─) represents one mutational step, and unsampled/intermediate haplotypes are shown by a white diamond (◊).

The topology of 12S rDNA, COII, and D-loop median-joining networks was similar: the presence of central haplotypes (hI and hII; h1 and h15; and hA, respectively) had a high frequency and was shared by several populations. Among these haplotypes, separated by 1–4 mutations, are low-frequency haplotypes unique to particular populations. The star-shaped network, mainly for COII and D-loop, was due to the low nucleotide diversity observed in the genes of *A. aureolatum* populations (0.0000–0.0048) (Table 1). No haplogroups were found (Figure 1).

### 2.3. Population Differentiation, Structuring, and Demographic Dynamics in A. aureolatum from Brazilian Spotted Fever Scenarios

With the exception of those from PR and SC, all populations showed significant genetic differentiation values for at least one marker in the pairwise *F*_ST_ analyzes (Table 2). Additionally, SF scenario-grouped *A. aureolatum* populations showed significant *F*_ST_ values for 12S rDNA and COII genes, suggesting that there was genetic divergence between populations of *A. aureolatum* that acted (SP) or not (PR, SC, RJ, and RS) as vectors of *R. rickettsii* (Table 3). However, Mantel test results indicated no significant correlation between genetic and geographic distances (12S rDNA: r = 0.1862, *p* = 0.276; COII: r = 0.1165, *p* = 0.298; D-loop: r = 0.1934, *p* = 0.268).

AMOVA analysis indicated that the majority of total molecular variation in *A. aureolatum* haplotypes occurred within populations (91.32% in 12S rDNA, 70.01% in COII, and 90.84% in D-loop) (Table 4). These results, plus the lack of significance in both the Φ statistic among groups (Table 4) and the Mantel tests, may indicate that no *A. aureolatum* population structure correlated with the current SF epidemiological scenarios.

Statistically significant values were obtained for the neutrality test of COII and/or D-loop markers in the PR, SC, RS, and SP *A. aureolatum* populations (Table 1). These results may be indicative of recent population expansion under both epidemiological scenarios studied.

### 2.4. Identification of Detected Rickettsiae

A total of five *A. aureolatum* samples collected from dogs in epidemiological scenario 1 (ES 1) (SC) (4749A) and epidemiological scenario 2 (ES 2) (SP) (6249H, I1, K, and R) amplified fragments of *glt*A and *htr*A genes from rickettsia (Table S4). BLAST analysis revealed 100% nucleotide identity rates with *Rickettsia felis* in samples from ES 1 and with *Rickettsia bellii* in samples from ES 2. In the phylogenetic reconstruction, these bacteria clustered with the same rickettsia species to which they showed similarity (Figure S1).

The majority of *A. aureolatum* samples identified with rickettsia in ES 1 and ES 2 belonged to the haplotypes both most frequent and shared by several populations (12S rDNA-hI, hII; COII-h1, h14, h15; and D-loop hA, hB). However, the same haplotype was detected with different rickettsia species, and the same rickettsia species was found in different *A. aureolatum* haplotypes (Table S4).

## 3. Discussion

This is the first study to provide a better understanding of SF epidemiology by integrating *A. aureolatum* population genetics data using mitochondrial genes and rickettsia infection. The number of identified haplotypes (7–17) was relatively small when compared to other *Amblyomma* species that are involved in the SF scenario in Brazil [12,13] (Tables S1–S3). This may be due to the low population density of *A. aureolatum* [3] and the high rate of endogamy previously reported for populations of this tick [14]. Of the analyzed markers, COII was the most sensitive for polymorphism detection, while 12S rDNA proved to be the most conserved in the *A. aureolatum* populations analyzed (Table 1 and Table S1–S3).

The *A. aureolatum* populations from RJ, RS, and PR had intermediate/non-sampled haplotypes (Figure 1B), suggesting that diversity in these populations may be greater than reported. The results of tick population genetics are strongly influenced by the sampling analyzed [10]. Accordingly, the greater number of exclusive haplotypes and higher diversity index values obtained in ES 1 populations compared to ES 2 (Table 1) may be associated with the larger area, collection period, populations/individuals, and different host species sampled (Tables S4 and S5). In contrast, individuals analyzed for ES 2 were all from the same location (Santo André/SP), and were all collected during the same period from dogs (Tables S4 and S5).

In general, *A. aureolatum* haplotypes differ from each other by few mutations (Figure 1). The presence of unique and low-frequency haplotypes to individual populations (Tables S1–S3) when added to the star-shaped network (COII and D-loop) (Figure 1B,C), as well as the high values of haplotype diversity, but low nucleotide diversity, and significant values in the neutrality tests for populations in ES 1 (SC, PR, and RS) and ES 2 (SP) (Table 1), may be indicative of a rapid and recent population expansion [15]. Climate and anthropogenic changes can influence tick demographic dynamics, which may in turn impact public health via increases in vector populations and, possibly, TBD bioagents [10]. This population expansion scenario has been observed previously for other tick species, such as *A. sculptum* in the Brazilian Cerrado biome [12]. The demographic pattern observed for *A. aureolatum* may be associated with Atlantic Forest fragmentation, which consequently leads to reductions in both the abundance and species richness of vertebrate hosts. This contrasts with the high density of domestic dogs, animals that can access the forest, then return to home infested with *A. aureolatum* to cause tick expansion on a local scale [7,8]. Additionally, the rapid population growth of this ixodid deserves attention since, under laboratory conditions, *A. aureolatum* adults infected with *R. rickettsii* can transmit this bacterium in about 10 min following initial skin attachment to a second host [16].

The presence of exclusive haplotypes for the ES 1 and ES 2 populations (Figure 1; Tables S1–S3) and the significant values in the pairwise (except for SC and PR) (Table 2) and *F*_ST_ group analyses (Table 3) together suggest genetic differentiation between *A. aureolatum* populations involved in different epidemiological scenarios analyzed. However, the following may indicate the presence of the gene flow and absence of *A. aureolatum* population structure between the different SF scenarios studied: the presence of haplotypes shared by all populations in all evaluated markers (Figure 1; Tables S1–S3); no haplogroup in the network (Figure 1); the AMOVA (most of the molecular variance occurred within studied populations); and the lack of significance in the Φ statistic results between groups (Table 4). In addition, the Mantel test confirms that genetic differentiation is not associated with geographical distances for the studied populations. The absence of population structure with low and non-significant pairwise *F*_ST_ values was also observed when comparing *A. aureolatum* from endemic and non-endemic BSF areas in SP [14]. Consequently, it is possible that other factors are responsible for the genetic differentiation observed in the analyzed populations that are not associated with the susceptibility of this ixodid to infection and vectoring of *R. rickettsii*.

Movements by *A. aureolatum* hosts could potentially influence the lack of structure observed between the tick populations, since this arthropod alone does not have a large dispersion capacity. Some bird groups that are infested by immature *A. aureolatum* may be the main agent responsible for maintaining the gene flow between different geographic populations of this ixodid, as canids (the main hosts for adult stage) and small rodents (hosts for larva and nymph) are likely to have a lower dispersion potential [14], mainly with the increase of Atlantic Forest fragmentation. Consequently, it is possible that structural and functional ecological connections between tick hosts could sustain gene flow between populations of this arthropod, explaining the observed patterns. The absence of a population structure associated with the movement of hosts has already been suggested for *A. sculptum* in Brazil by [11,12], who considered that low parasite specificity, availability of preferred hosts in degraded areas, and the human transportation of infested hosts (e.g., horses) were the main agents responsible for the maintenance of the gene flow between *A. sculptum* populations.

Although *A. aureolatum* and *A. ovale* have some similar ecological and morphological characteristics, mainly in areas where they occur in sympathy, the results of population genetics obtained for the two species were different. While *A. aureolatum* showed an absence of a population structure [14] associated with population growth, *A. ovale* populations were structured, possibly associated with ecological factors and geographical distances between the studied populations [13]. These differences are not unexpected, as ticks are a highly diverse group of ectoparasites, and each species may have specific characteristics relating to life history strategies and/or evolutionary pressures that can result in distinct genetic signatures [10].

Estimates of genetic structure and dispersal in ticks may serve as a surrogate for dispersal estimates of tick-borne pathogens [10]. However, no relationship was observed between *A. aureolatum* genetic patterns and the different SF scenarios in Brazil. Thus, other factors, such as ecology and environment, may influence the action of *A. aureolatum* as a *R. rickettsii* vector in certain areas and not in others. However, the absence of population structure associated with anthropic actions that have been causing environmental changes, such as the fragmentation of the Atlantic Forest, as observed for the endemic area for SF associated with *A. aureolatum* in SP [8], may cause this tick over time to become an important vector of *R. rickettsii* in other degraded areas of the Atlantic Forest throughout the country. Thus, it can generate new outbreaks of SF cases with an impact on public health. Although *A. aureolatum* and *A. sculptum* are vectors of *R. rickettsii*, the first one is more susceptible to this infection [17].

Detection of *R. bellii* during environmental surveillance for SF occurred in an area with previous records of BSF cases in SP (Figure S1, Table S4). There are previous reports of this bacterium in *A. aureolatum* in the Brazilian states of SP, RJ, and Espírito Santo (e.g., [7,18,19]). Even though the pathogenicity of this rickettsia to humans is unknown, it can play an important role in the epidemiology of rickettsial diseases by inhibiting the maintenance of other pathogenic rickettsia species in the tick [20,21]. Consequently, the detection of *R. bellii* in the metropolitan region of SP is important, since, in that area, *A. aureolatum* has been identified as the main vector of *R. rickettsii* [1].

*Rickettsia felis* is the bioagent of flea-borne spotted fever (FBSF), an emerging disease. However, basic knowledge of its biology and FBSF epidemiology remains incipient in Brazil. The presence of a confirmed FBSF case in the country [22] highlights the need for research related to this bacterium. There is a previous report of *R. felis* in *A. aureolatum* in SP [7]. However, this is the first detection of this rickettsia in this tick for SC. This infection may be due to a simultaneous infestation of the dog by both *A. aureolatum-* and *R. felis*-infected fleas (*Ctenocephalides* spp.), which are admittedly vectors of this rickettsia. However, we cannot rule out the possibility of *A. aureolatum* participation in the *R. felis* cycle. Although this tick acts as an important vector of *R. rickettsii* in the metropolitan region of SP [1], it may be that in other areas of the country this ixodid has a relevant role in the cycle of other rickettsia. In southern Brazil, *A. aureolatum* is abundant [2,23], although there is, so far, no confirmation of infection by *R. rickettsii* [1]. The detection of *R. felis*, a bioagent associated with mild SF cases, in *A. aureolatum* from SC, a region with confirmed cases of but no deaths from SF during SF case investigation, may indicate the participation of this tick in different eco-epidemiological scenarios of the disease in Brazil.

Thus, it is possible that the role of *A. aureolatum* as a potential vector for rickettsia in southern Brazil, where this ixodid has already been detected to be infected with the *Rickettsia parkeri* strain Atlantic rainforest [24], *Rickettsia parkeri sensu stricto* [4,6], and *Rickettsia asembonensis* [6], is different from what is known to-date for the metropolitan region of SP [1]. Further studies are needed to assess whether *A. aureolatum* is also capable of transmitting *R. felis*. In addition, evaluating the implications associated with this rickettsia in the country, as well as the participation of *A. aureolatum*, should be investigated.

Although *A. aureolatum* is an efficient *R. rickettsii* vector [1,17], the absence of this infection in the analyzed samples, even from BSF endemic area in SP, does not allow us to exclude the existence of this bacterium and/or another rickettsia species infecting the tick populations within the studied locations. Low infection rates (1–10%) of *R. rickettsii* are reported in *A. aureolatum* in the wild, possibly related with the deleterious effect that this bioagent has on this tick [7,18].

The lack of a relationship between tick haplotypes and rickettsia detected in *A. aureolatum* may be associated with the lack of genetic structuring among the analyzed populations of this arthropod. A previous study also detected weak differentiation and a lack of structure between *A. aureolatum* populations in endemic and non-endemic BSF areas in SP, which did not support the hypothesis that analyzed genetic characteristics of *A. aureolatum* populations would be responsible for the presence or absence of *R. rickettsii* in these areas of SP [14].

For a better understanding of *A. aureolatum* participation in SF epidemiological scenarios in Brazil, it may be necessary to analyze other genetic regions of this tick that are under strong selective pressure related to forms of natural resistance to infection by *R. rickettsii* and/or other rickettsia [14]. In addition, ecological and environmental factors can influence the presence or absence of rickettsia and their amplifying hosts [7]. Atlantic Forest fragmentation is correlated with a high risk of BSF associated with *A. aureolatum* in SP [8], since deforestation can result in habitat loss and create environmental conditions unfavorable to the maintenance of populations of large carnivores, and this often makes domestic dogs the only available host for the adult stage of this ixodid. With access to the forest and without competition from wild carnivores, dogs can roam freely in the vegetation, increasing the chance of *A. aureolatum* parasitism. Upon returning parasitized to human habitations, these animals increase the risk of human infection with rickettsia [8]. Future studies that seek a broader understanding of the epidemic and enzootic cycles of SF involving *A. aureolatum* should analyze samples of the different stages of this ixodid from birds, small rodents, and wild canids. For future phylogenetic and population analyzes of the tick, other markers can be used, such as the 16S rDNA that is commonly used for the group, which enable a greater understanding of the inter- and intraspecific relationships of ixodids in the different epidemiological scenarios of SF.

The absence of structure among analyzed *A. aureolatum* populations associated with the expansion of this vector in PR, SC, RS, and SP requires public health attention, since population growth of this ixodid, a competent vector for *R. rickettsii*, could potentially cause enhanced pathogen dispersion [10], thus generating new foci of SF. The integrated use of tick population genetics data, the presence of pathogens, transcriptome and microbiome studies, and ecological analysis can be useful tools for a broader understanding of the processes surrounding, and perhaps regulating, TBDs in Brazil.

## 4. Materials and Methods

### 4.1. Sampling and Taxonomy

**Figure 2:**
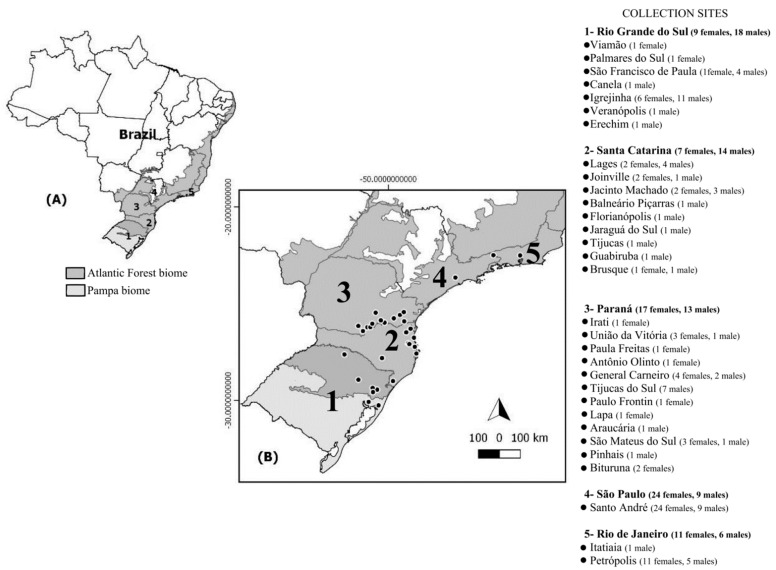
(**A**) Map of Brazil with indication of studied states (1–5) and respective biomes; (**B**) number of individuals sampled at each collection sites (•) for the 128 *Amblyomma aureolatum* specimens analyzed from Brazil.

In total, 128 (68 females and 60 males) *A. aureolatum* were collected from vertebrate hosts (*Canis familiaris* (102), *Felis catus* (9), *Homo sapiens* (9), *Bos taurus* (1), and *Nasua nasua* (1)) and the environment (six human habitations), between 2012 to 2017 during SF-focused investigations and environmental surveillance in Brazil in localities with altitudes varying from 2–1648 m by teams from five state departments of health (Tables S4 and S5). The samples came from states where there is no evidence of *A. aureolatum* involvement in the BSF epidemic cycle (PR, SC, RS, and RJ; ES 1) and from an area where this ixodid demonstrably participates in the BSF cycle as the vector of *R. rickettsii* (metropolitan region of SP; ES 2) (Figure 2). Obtained *A. aureolatum* were stored in isopropyl alcohol, then sent to the Laboratory of the National Reference of Rickettsial Vectors, where they were morphologically identified [23] and analyzed individually with molecular biology techniques. The states and epidemiological scenarios of tick collection were used to determine populations in genetic analyzes.

### 4.2. DNA Extraction, Amplification, and Sequencing

In all samples, DNA was extracted using the saturated sodium chloride solution protocol [25]. For *A. aureolatum* population analysis, a set of mitochondrial gene fragments (12S rDNA [26], D-loop [27], and COII [11]) was amplified by a polymerase chain reaction (PCR) following previously established protocols [11].

Rickettsia-infected ticks were detected by PCR screening for the rickettsial *glt*A (CS2-78/CS2-323) [28], *omp*A (Rr l90.70p/Rr l90.602n) [29], *omp*B (120.M59/120.807) [30], and *htr*A (nested PCR, first-round primers 17k-5/17k-3) [31]; (second round primers 17kD1/17kD2) [32] genes. In these amplifications, DNA-free reactions was used as negative control, with *R. rickettsii* DNA as positive control.

PCR products were visualized by electrophoresis in 2% agarose gel stained with ethidium bromide and purified using the Wizard^®^SV Gel and PCR Clean-Up System kit (Promega Corp., Madison, WI, USA), following manufacturer protocols. DNA was sequenced in both directions, applying the same PCR primers and the BigDye Terminator™ version 3.1 Cycle Sequencing^®^ Kit (Applied Biosystems, Foster City, CA, USA) on an automated ABI 3730xl DNA analyser (Applied Biosystems, Foster City, CA, USA,). Sequences from tick haplotypes and from identified rickettsia were deposited in GenBank (accession numbers MF175654-8, MF175680-91, MF175738-47, MF175769-72, MF175786-8, MT578021-31) (Table S4).

### 4.3. Sequence Editing, Identification, and Alignment

The obtained sequences were assembled and edited with CHROMASPRO, version 1.5 (Technelysium Pty Ltd., Tewantin, QLD, Australia), and subjected to BLASTn analyses (https://blast.ncbi.nlm.nih.gov/Blast.cgi, accessed on 16 February 2021) to infer closest similarities with other organisms available in GenBank. Multiple alignments were conducted using the ClustalW algorithm, and then checked manually. Protein coding genes were translated into amino acids where no stop codon was observed.

### 4.4. Amblyomma aureolatum Population Genetics Analysis

Haplotype definition (considered distinct when differing by at least one base pair), polymorphic sites (*s*), number of haplotypes (*n*), both haplotype (h) and nucleotide diversity (nd), and the average number of nucleotide differences (k) were calculated in ARLEQUIN software, version 3.5.2.2 [33].

Analysis of molecular variance (AMOVA) [34] and pairwise *F*_ST_ structuring values were used to estimate molecular genetic differentiation between *A. aureolatum* populations. Calculations of Φ statistics indices were based on AMOVA values. Mantel tests analyzed the association between genetic (using *F*_ST_ values) and geographic distances (from Google Earth). Additionally, *F*_ST_ and AMOVA tests were determined hierarchical supra-population levels (Group I and II) to check if *A. aureolatum* genetic differentiation and population structure varied between SF scenarios, where: Group I-samples from ES 1 (PR, SC, RJ and RS) and Group II-samples from ES 2 (metropolitan area of SP). These analyzes were performed using ARLEQUIN, with 1000 repeats each.

The Tajima’s *D* [15], Fu’s *Fs* [35] and R_2_ [36] neutrality tests were performed to investigate the hypothesis of neutral mutation and population balance. To estimate the indices, ARLEQUIN software was used for Tajima’s *D* and Fu’s *Fs*, and DNASP v. 6.12.03 [37] for the R_2_ test, with 1000 permutations in each case.

A haplotype network was constructed for each marker using NETWORK 10.0.0.0 software (Fluxus Technology Ltd., Cambridge, MA, U.K.), combined with a median joining algorithm [38].

### 4.5. Phylogenetic Relationships of Detected Rickettsiae

A concatenated (*glt*A + *htr*A, 1383 bp) Maximum-Likelihood phylogeny was inferred using PhyML software Version 3.0 [39], with evolutionary model GTR+G indicated by MEGA 6.0, through BIC. Alignments were concatenated with Seaview software [40]. Rickettsiae sequences generated in this study were used in the phylogenetic reconstruction, plus those available in GenBank. The reliability of the tree topology was estimated with an approximate likelihood ratio test (aLRT) with 1000 replicas.

## Figures and Tables

**Table 1:** Summary of 12S rDNA, cytochrome oxidase subunit II, and D-loop haplotype distribution, variability, genetic diversity, and demographic history measures of *Amblyomma aureolatum* populations analyzed from different spotted fever scenarios in Brazil.

Gene/Region	Epidemiological Scenario	Population	Sample Size	*n*	*s*	k ± SD	h ± SD	nd ± SD	Fu’s *Fs* (*p*-Value)	Tajima’s *D* (*p*-Value)	R2 (*p*-Value)
12S rDNA	Group I	Paraná	21	4	4	1.22 ± 0.81	0.614 ± 0.062	0.0033 ± 0.0024	0.516 (0.62)	0.275 (0.65)	0.148 (0.56)
		Santa Catarina	19	2	2	1.06 ± 0.73	0.526 ± 0.040	0.0028 ± 0.0022	3.031 (0.96)	2.006 (0.99)	0.263 (1.00)
		Rio de Janeiro	4	1	0	0.00 ± 0.00	0.000 ± 0.000	0.0000 ± 0.0000	N.A.	0.000 (1.00)	N.A.
		Rio Grande do Sul	17	5	5	1.71 ± 1.05	0.787 ± 0.059	0.0046 ± 0.0032	0.007 (0.53)	0.495 (0.71)	0.166 (0.68)
	Group II	São Paulo	29	2	2	0.83 ± 0.61	0.414 ± 0.077	0.0022 ± 0.0018	2.787 (0.95)	1.293 (0.90)	0.207 (0.93)
COII	Group I	Paraná	16	7	8	1.31 ± 0.86	0.718 ± 0.128	0.0027 ± 0.0020	−3.113 (0.00) *	−1.649 (0.04) *	0.113 (0.06)
		Santa Catarina	13	5	3	0.73 ± 0.58	0.533 ± 0.142	0.0015 ± 0.0013	−2.582 (0.01) *	−1.471 (0.03) *	0.147 (0.17)
		Rio de Janeiro	4	2	2	1.34 ± 1.03	0.667 ± 0.204	0.0027 ± 0.0025	1.530 (0.86)	1.893 (0.95)	0.333 (0.44)
		Rio Grande do Sul	10	7	10	2.35 ± 1.39	0.911 ± 0.077	0.0048 ± 0.0032	−2.785 (0.02)	−1.507 (0.09)	0.098 (0.00) *
	Group II	São Paulo	15	5	4	0.76 ± 0.59	0.638 ± 0.129	0.0016 ± 0.0014	−2.233 (0.07)	−1.220 (0.08)	0.105 (0.00) *
D-loop	Group I	Paraná	24	6	6	0.80 ± 0.60	0.601 ± 0.108	0.0017 ± 0.0015	−2.678 (0.01) *	−1.524 (0.03) *	0.086 (0.06)
		Santa Catarina	17	4	3	0.35 ± 0.36	0.331 ± 0.143	0.0007 ± 0.0009	−2.527 (0.00) *	−1.706 (0.01) *	0.127 (014)
		Rio de Janeiro	16	4	3	0.58 ± 0.49	0.517 ± 0.132	0.0013 ± 0.0012	−1.478 (0.06)	−1.055 (0.16)	0.123 (0.11)
		Rio Grande do Sul	26	3	5	1.93 ± 1.13	0.542 ± 0.075	0.0042 ± 0.0028	3.520 (0.97)	1.319 (0.87)	0.192 (0.92)
	Group II	São Paulo	27	6	6	0.65 ± 0.52	0.399 ± 0.117	0.0014 ± 0.0013	−3.205 (0.01) *	−1.718 (0.01) *	0.080 (0.08)

*n*, Number of haplotypes; *s*, number of polymorphic sites; k, mean number of pairwise differences; h, gene/haplotype diversity; nd, nucleotide diversity/loci; SD, standard deviation; *p*, statistical *p*-value; N.A., not applicable; R_2_, Ramos–Onsins and Rozas R_2_ statistic; *p* < 0.05, except for Fu’s *Fs p* < 0.02; * significant; COII, cytochrome oxidase subunit II; Group I, populations where there was no evidence of *Amblyomma aureolatum* participation in the Brazilian spotted fever scenario; Group II, only population in which *Amblyomma aureolatum* was a proven vector of *Rickettsia rickettsii*.

**Table 2:** Pairwise comparisons for genetic differentiation (*F*_ST_) estimated for all *Amblyomma aureolatum* populations sampled from different spotted fever scenarios in Brazil.

Population	Marker	Paraná	Santa Catarina	Rio de Janeiro	Rio Grande do Sul
Santa Catarina	12S rDNA	−0.03027			
	COII	−0.04289			
	D-loop	−0.01711			
Rio de Janeiro	12S rDNA	0.32037	0.25709		
	COII	0.43171 *	0.54683 *		
	D-loop	−0.02385	0.00731		
Rio Grande do Sul	12S rDNA	−0.01408	−0.01599	0.21181	
	COII	0.31469 *	0.35290 *	0.12080	
	D-loop	0.18499 *	0.20231 *	0.18353 *	
São Paulo ^♦^	12S rDNA	0.01039	0.08547	0.55767 *	0.05795
	COII	0.41611*	0.48016 *	0.28403 *	0.05003
	D-loop	−0.01457	−0.01774	−0.01781	0.21033 *

COII, cytochrome oxidase subunit II; * significant (*p* < 0.05); ^♦^ only population where *Amblyomma aureolatum* was a proven vector of *Rickettsia rickettsii*.

**Table 3:** Pairwise comparisons for genetic differentiation (*F*_ST_) estimated between groups of *Amblyomma aureolatum* populations from different spotted fever scenarios in Brazil.

Population	Marker	Group II
Group I	12S rDNA	0.06858 *
	COII	0.17215 *
	D-loop	0.02085

Group I—Paraná, Santa Catarina, Rio de Janeiro, and Rio Grande do Sul; Group II—São Paulo (only population where *Amblyomma aureolatum* was a proven vector of *Rickettsia rickettsii*); COII, cytochrome oxidase subunit II; * significant (*p* < 0.05).

**Table 4:** Analysis of molecular variance (AMOVA) for the analyzed genetic sequences of the 12S rDNA, cytochrome oxidase subunit II, and D-loop markers of groups and studied Brazilian populations of *Amblyomma aureolatum*.

		Analyzed Marker					
AMOVA	(Φ Statistic)	12S rDNA		COII		D-Loop	
Among groups	(Φ_CT_)	3.80%	(0.038)	−1.37%	(−0.014)	−9.14%	(−0.091)
Among populations within groups	(Φ_SC_)	4.88%	(0.051)	31.36%	(0.309 *)	18.30%	(0.168 *)
Within populations	(Φ_ST_)	91.32%	(0.087*)	70.01%	(0.300 *)	90.84%	(0.092 *)

Group I—Paraná, Santa Catarina, Rio de Janeiro, and Rio Grande do Sul; Group II—São Paulo (only population where *Amblyomma aureolatum* was a proven vector of *Rickettsia rickettsii*); COII, cytochrome oxidase subunit II; * significant (*p* < 0.05).

## Data Availability

The data that support the findings of this study are available within the article, in Supplementary Materials and in GenBank at https://www.ncbi.nlm.nih.gov/genbank/, accessed on 11 March 2021, accession numbers: MF175654-8, MF175680-91, MF175738-47, MF175769-72, MF175786-8, MT578021-31.

## Supplementary Materials

The following are available online at https://www.mdpi.com/article/10.3390/pathogens10091146/s1, Figure S1: Concatenated phylogeny of rickettsia *glt*A and *htr*A genes fragment (1013 + 370 bp) detected in *Amblyomma aureolatum* in Brazil, inferred by Maximum Likelihood analysis with evolution model GTR+G. Numbers on the branches represent support values (70% cut-off), Table S1: Distribution and frequency of 12S rDNA haplotypes identified in the populations of *Amblyomma aureolatum* analyzed from Brazil, Table S2: Distribution and frequency of cytochrome oxidase subunit II haplotypes identified in the populations of *Amblyomma aureolatum* analyzed from Brazil, Table S3: Distribution and frequency of D-loop haplotypes identified in the populations of *Amblyomma aureolatum* analyzed from Brazil, Table S4: Sample data, haplotype and associated GenBank accession numbers for *Amblyomma aureolatum* 12S rDNA, cytochrome oxidase subunit II (COII), D-loop and rickettsia sequences analyzed from Brazil, Table S5: Collection data for *Amblyomma aureolatum* samples analyzed from Brazil.
