# Supplementary material for: Amblyomma aureolatum Genetic Diversity and Population Dynamics Are Not Related to Spotted Fever Epidemiological Scenarios in Brazil
Source: Pathogens. 2021 Sep 6;10(9):1146. doi: 10.3390/pathogens10091146 (PMC8469259; doi:10.3390/pathogens10091146)
Supplement: Supplementary file 1 [file pathogens-10-01146-s001.zip › Figure S1.pdf]

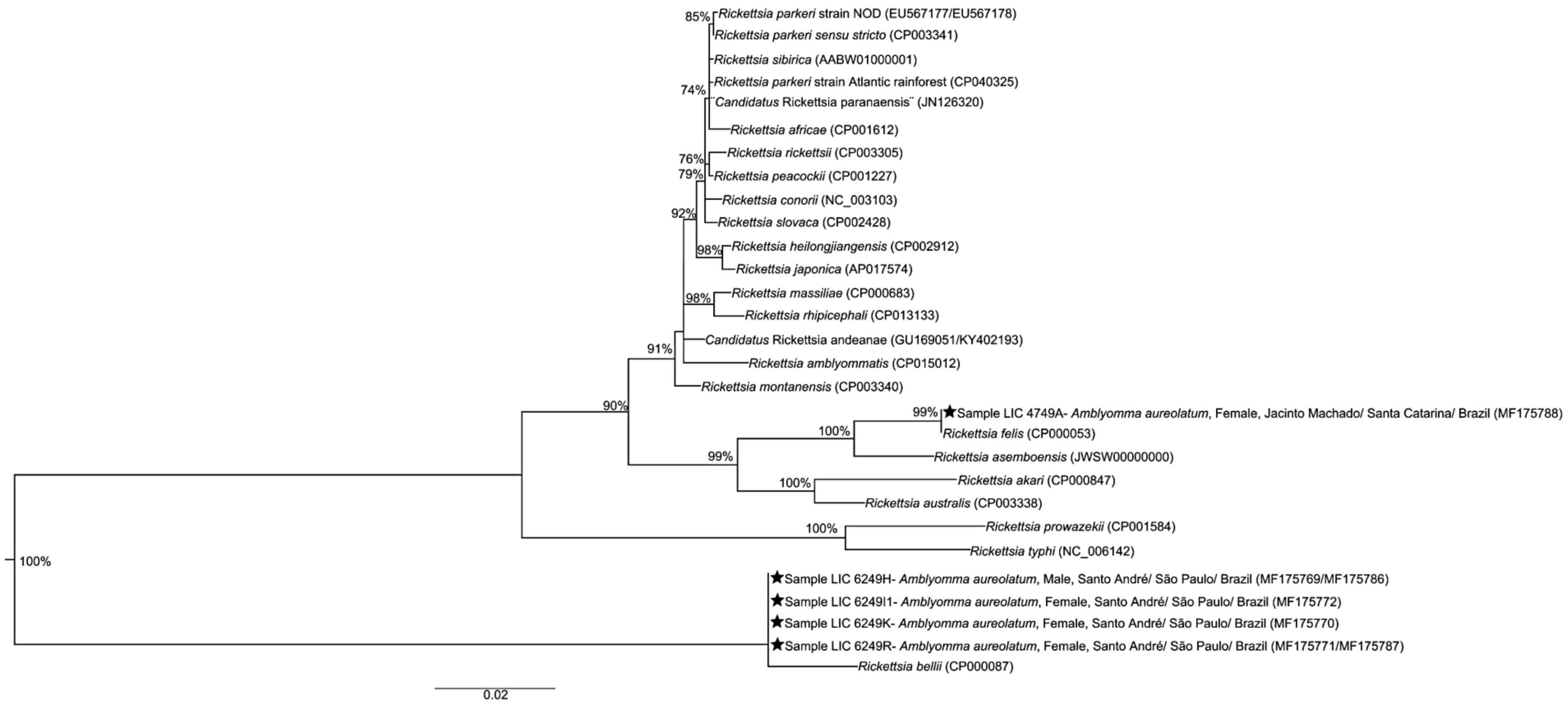

Figure S1. Concatenated phylogeny of rickettsia *glt*A and *htr*A genes fragment (1013+ 370 bp) detected in *Amblyomma aureolatum* in Brazil, inferred by Maximum Likelihood analysis with evolution model GTR+G. Numbers on the branches represent support values (70% cut-off).
