# Supplementary material for: Amblyomma aureolatum Genetic Diversity and Population Dynamics Are Not Related to Spotted Fever Epidemiological Scenarios in Brazil
Source: Pathogens. 2021 Sep 6;10(9):1146. doi: 10.3390/pathogens10091146 (PMC8469259; doi:10.3390/pathogens10091146)
Supplement: Supplementary file 1 [file pathogens-10-01146-s001.zip › Table S1.pdf]

Table S1. Distribution and frequency of 12S rDNA haplotypes identified in the populations of *Amblyomma aureolatum* analyzed from Brazil**.**

Epidemiological scenario	Populations	*Ns*	12S rDNA Haplotypes (371 bp)						
			hI	hII	hIII	hIV	hV	hVI	hVII
Group I	Paraná	21	10	9				1	1
	Santa Catarina	19	9	10					
	Rio de Janeiro	4		4					
	Rio Grande do Sul	17	6	5	1	2	3		
Group II	São Paulo	29	21	8					
	Total	90	46	36	1	2	3	1	1
	Frequency (%)	100.00	51.12	40.00	1.11	2.22	3.33	1.11	1.11

Ns = number of sequences; Group I, populations where there is no evidence of *Amblyomma aureolatum* participation in the Brazilian Spotted Fever scenario; Group II, Only population in which *Amblyomma aureolatum* is a proven vector of *Rickettsia rickettsii* .
