# Supplementary material for: Amblyomma aureolatum Genetic Diversity and Population Dynamics Are Not Related to Spotted Fever Epidemiological Scenarios in Brazil
Source: Pathogens. 2021 Sep 6;10(9):1146. doi: 10.3390/pathogens10091146 (PMC8469259; doi:10.3390/pathogens10091146)
Supplement: Supplementary file 1 [file pathogens-10-01146-s001.zip › Table S2.pdf]

Table S2. Distribution and frequency of cytochrome oxidase subunit II haplotypes identified in the populations of *Amblyomma aureolatum* analyzed from Brazil**.**

Epidemiological scenario	Populations	*Ns*	Cytochrome oxidase subunit II Haplotypes (487 bp)																
			h1	h2	h3	h4	h5	h6	h7	h8	h9	h10	h11	h12	h13	h14	h15	h16	h17
Group I	Paraná	16	9			2							1	1	1	1	1		
	Santa Catarina	13	9	1	1	1											1		
	Rio de Janeiro	4					2										2		
	Rio Grande do Sul	10								1	1	1				2	3	1	1
Group II	São Paulo	15		1				2	1							2	9		
	Total	58	18	2	1	3	2	2	1	1	1	1	1	1	1	5	16	1	1
	Frequency (%)	100.00	31.04	3.45	1.72	5.18	3.45	3.45	1.72	1.72	1.72	1.72	1.72	1.72	1.72	8.63	27.60	1.72	1.72

Ns = number of sequences; Group I, populations where there is no evidence of *Amblyomma aureolatum* participation in the Brazilian Spotted Fever scenario; Group II, Only population in which *Amblyomma aureolatum* is a proven vector of *Rickettsia rickettsii* .
