# Supplementary material for: Amblyomma aureolatum Genetic Diversity and Population Dynamics Are Not Related to Spotted Fever Epidemiological Scenarios in Brazil
Source: Pathogens. 2021 Sep 6;10(9):1146. doi: 10.3390/pathogens10091146 (PMC8469259; doi:10.3390/pathogens10091146)
Supplement: Supplementary file 1 [file pathogens-10-01146-s001.zip › Table S3.pdf]

Table S3. Distribution and frequency of D-loop haplotypes identified in the populations of *Amblyomma aureolatum* analyzed from Brazil.

Epidemiological scenario	Populations	*Ns*	D-loop Haplotypes (458 bp)													
			hA	hB	hC	hD	hE	hF	hG	hH	hI	hJ	hK	hL	hM	hN
Group I	Paraná	24	15	3	2			2							1	1
	Santa Catarina	17	14	1	1	1										
	Rio de Janeiro	16	11	3			1	1								
	Rio Grande do Sul	26	16										8	2		
Group II	São Paulo	27	21	1					1	1	2	1				
	Total	110	77	8	3	1	1	3	1	1	2	1	8	2	1	1
	Frequency (%)	100.00	70.01	7.27	2.73	0.91	0.91	2.73	0.91	0.91	1.81	0.91	7.27	1.81	0.91	0.91

Ns = number of sequences; Group I, populations where there is no evidence of *Amblyomma aureolatum* participation in the Brazilian Spotted Fever scenario; Group II, Only population in which *Amblyomma aureolatum* is a proven vector of *Rickettsia rickettsii* .
