# Supplementary material for: Amblyomma aureolatum Genetic Diversity and Population Dynamics Are Not Related to Spotted Fever Epidemiological Scenarios in Brazil
Source: Pathogens. 2021 Sep 6;10(9):1146. doi: 10.3390/pathogens10091146 (PMC8469259; doi:10.3390/pathogens10091146)
Supplement: Supplementary file 1 [file pathogens-10-01146-s001.zip › Table S4.pdf]

Table S4. Sample data, haplotype and associated GenBank accession numbers for *Amblyomma aureolatum* 12S rDNA, cytochrome oxidase subunit II (COII), D-loop and rickettsia sequences analyzed from Brazil.

Sample number	Tick sex	Host	Year collection	State	Localities	Altitude	Presence of rickettsia (Amplified gene) (GenBank accession number)	12S rDNA Haplotype (GenBank accession number)	COII Haplotype (GenBank accession numbers)	D-loop Haplotypes (GenBank accession numbers)
7402	Female	*Homo sapiens*	2015	Paraná	Irati	836 meters	Negative	hII (MF175655)	h15 (MT578027)	hB (MF175681)
8108	Female	*Canis familiaris*	2016	Paraná	União da Vitória	752 meters	Negative	hVI (MT578021)	h11 (MT578023)	hA (MF175680)
8109	Female	*Canis familiaris*	2016	Paraná	Paula Freitas	793 meters	Negative	hVII (MT578022)	–	hM (MT578030)
8181	Female	*Canis familiaris*	2016	Paraná	Antônio Olinto	817 meters	Negative	hI (MF175654)	–	hA (MF175680)
8193	Female	*Canis familiaris*	2016	Paraná	União da Vitória	752 meters	Negative	hI (MF175654)	h12 (MT578024)	hA (MF175680)
8194	Female	Environment (House)	2016	Paraná	General Carneiro	983 meters	Negative	hII (MF175655)	h1 (MF175738)	hC (MF175682)
8195	Female	*Canis familiaris*	2016	Paraná	General Carneiro	983 meters	Negative	hI (MF175654)	h4 (MF175741)	hA (MF175680)
8499	Male	*Homo sapiens*	2017	Paraná	Tijucas do Sul	940 meters	Negative	hI (MF175654)	h1 (MF175738)	hA (MF175680)
8526	Female	*Homo sapiens*	2017	Paraná	Paulo Frontin	778 meters	Negative	hI (MF175654)	–	hA (MF175680)
8529	Female	Environment (House)	2017	Paraná	General Carneiro	983 meters	Negative	–	–	hA (MF175680)
8530	Male	*Felis catus*	2017	Paraná	General Carneiro	983 meters	Negative	hII (MF175655)	–	–
8533	Female	*Homo sapiens*	2017	Paraná	Lapa	908 meters	Negative	hII (MF175655)	h1 (MF175738)	hF (MF175685)
8534	Male	*Canis familiaris*	2017	Paraná	Araúcária	897 meters	Negative	–	–	hB (MF175681)
8632	Female	Environment (House)	2017	Paraná	São Mateus do Sul	761 meters	Negative	hI (MF175654)	h14 (MT578026)	hA (MF175680)
8637	Male	*Canis familiaris*	2017	Paraná	Pinhais	893 meters	Negative	hI (MF175654)	–	hA (MF175680)
8107A	Female	*Canis familiaris*	2016	Paraná	São Mateus do Sul	761 meters	Negative	hII (MF175655)	–	hF (MF175685)
8107B	Male	*Canis familiaris*	2016	Paraná	São Mateus do Sul	761 meters	Negative	hI (MF175654)	h1 (MF175738)	hA (MF175680)
8524A	Female	*Felis catus*	2017	Paraná	Bituruna	900 meters	Negative	–	h1 (MF175738)	hN (MT578031)
8524B	Female	*Felis catus*	2017	Paraná	Bituruna	900 meters	Negative	–	h1 (MF175738)	–
8531A	Female	*Canis familiaris*	2017	Paraná	General Carneiro	983 meters	Negative	hII (MF175655)	h13 (MT578025)	–
8531B	Male	*Canis familiaris*	2017	Paraná	General Carneiro	983 meters	Negative	hII (MF175655)	h1 (MF175738)	hA (MF175680)
8547A	Male	*Canis familiaris*	2017	Paraná	Tijucas do Sul	940 meters	Negative	hI (MF175654)	–	–
8547B	Male	*Canis familiaris*	2017	Paraná	Tijucas do Sul	940 meters	Negative	hII (MF175655)	–	hA (MF175680)
8548A	Male	*Canis familiaris*	2017	Paraná	Tijucas do Sul	940 meters	Negative	hI (MF175654)	h4 (MF175741)	hA (MF175680)
8548B	Male	*Canis familiaris*	2017	Paraná	Tijucas do Sul	940 meters	Negative	hII (MF175655)	h1 (MF175738)	hB (MF175681)
8548C	Male	*Canis familiaris*	2017	Paraná	Tijucas do Sul	940 meters	Negative	–	–	hA (MF175680)
8548D	Male	*Canis familiaris*	2017	Paraná	Tijucas do Sul	940 meters	Negative	–	h1 (MF175738)	–
8645A	Female	*Canis familiaris*	2017	Paraná	União da Vitória	752 meters	Negative	–	–	hC (MF175682)
8645B	Male	*Canis familiaris*	2017	Paraná	União da Vitória	752 meters	Negative	–	–	hA (MF175680)
4724B	Male	*Canis familiaris*	2013	Santa Catarina	Lages	930 meters	Negative	hI (MF175654)	h4 (MF175741)	hA (MF175680)
4724C	Male	*Canis familiaris*	2013	Santa Catarina	Lages	930 meters	Negative	hI (MF175654)	h1 (MF175738)	–
4724D	Male	*Canis familiaris*	2013	Santa Catarina	Lages	930 meters	Negative	hI (MF175654)	h1 (MF175738)	–

COII, cytochrome oxidase subunit II.

Table S4. Sample data, haplotype and associated GenBank accession numbers for *Amblyomma aureolatum* 12S rDNA, cytochrome oxidase subunit II (COII), D-loop and rickettsia sequences analyzed from Brazil (continuation).

Sample number	Tick sex	Host	Year collection	State	Localities	Altitude	Presence of rickettsia (Amplified gene) (GenBank accession number)	12S rDNA Haplotype (GenBank accession number)	COII Haplotype (GenBank accession numbers)	D-loop Haplotypes (GenBank accession numbers)
4725D	Male	*Nasua nasua*	2013	Santa Catarina	Joinville	4,5 meters	Negative	hI (MF175654)	–	–
4749A	Female	*Canis familiaris*	2013	Santa Catarina	Jacinto Machado	28 meters	*Rickettsia felis* (*htr*A) (MF175788)	hI (MF175654)	h1 (MF175738)	hA (MF175680)
4749B	Female	*Canis familiaris*	2013	Santa Catarina	Jacinto Machado	28 meters	Negative	hII (MF175655)	h1 (MF175738)	–
4749E	Male	*Canis familiaris*	2013	Santa Catarina	Jacinto Machado	28 meters	Negative	hII (MF175655)	h3 (MF175740)	hA (MF175680)
4749F	Male	*Canis familiaris*	2013	Santa Catarina	Jacinto Machado	28 meters	Negative	hII (MF175655)	h1 (MF175738)	hA (MF175680)
4749G	Male	*Canis familiaris*	2013	Santa Catarina	Jacinto Machado	28 meters	Negative	hII (MF175655)	h1 (MF175738)	hA (MF175680)
5378	Female	*Canis familiaris*	2014	Santa Catarina	Lages	930 meters	Negative	–	–	hA (MF175680)
5379	Female	*Canis familiaris*	2014	Santa Catarina	Lages	930 meters	Negative	–	–	hA (MF175680)
6461	Male	*Canis familiaris*	2014	Santa Catarina	Balneário Piçarras	18 meters	Negative	hII (MF175655)		hA (MF175680)
6465	Male	*Homo sapiens*	2014	Santa Catarina	Florianópolis	2,74 meters	Negative	hII (MF175655)	–	hA (MF175680)
6480	Male	*Bos taurus*	2014	Santa Catarina	Lages	930 meters	Negative	hI (MF175654)	–	hA (MF175680)
6479A	Female	*Canis familiaris*	2014	Santa Catarina	Joinville	4,5 meters	Negative	hI (MF175654)	–	hA (MF175680)
6479B	Female	*Canis familiaris*	2014	Santa Catarina	Joinville	4,5 meters	Negative	hII (MF175655)	–	hD (MF175683)
7021A	Male	*Canis familiaris*	2015	Santa Catarina	Jaraguá do Sul	30 meters	Negative	hII (MF175655)	h15 (MT578027)	hC (MF175682)
7380A	Male	*Canis familiaris*	2015	Santa Catarina	Tijucas	2 meters	Negative	hI (MF175654)	h2 (MF175739)	hB (MF175681)
8571D	Male	*Canis familiaris*	2017	Santa Catarina	Guabiruba	60 meters	Negative	hI (MF175654)	h1 (MF175738)	hA (MF175680)
8572B	Female	*Homo sapiens*	2017	Santa Catarina	Brusque	21 meters	Negative	hII (MF175655)	h1 (MF175738)	hA (MF175680)
8572C	Male	*Homo sapiens*	2017	Santa Catarina	Brusque	21 meters	Negative	hII (MF175655)	h1 (MF175738)	hA (MF175680)
4207A	Female	*Canis familiaris*	2012	Rio Grande do Sul	Viamão	9 meters	Negative	hIV (MF175657)	–	–
5526A	Female	*Canis familiaris*	2013	Rio Grande do Sul	São Francisco de Paula	889 meters	Negative	–	–	hA (MF175680)
5526B	Male	*Canis familiaris*	2013	Rio Grande do Sul	São Francisco de Paula	889 meters	Negative	–	–	hA (MF175680)
5526C	Male	*Canis familiaris*	2013	Rio Grande do Sul	São Francisco de Paula	931 meters	Negative	hV (MF175658)	–	hK (MF175690)
5526D	Male	*Canis familiaris*	2013	Rio Grande do Sul	São Francisco de Paula	889 meters	Negative	hI (MF175654)	h15 (MT578027)	hA (MF175680)
5526E	Male	*Canis familiaris*	2013	Rio Grande do Sul	São Francisco de Paula	889 meters	Negative	hI (MF175654)	h15 (MT578027)	hL (MF175691)
5527	Male	*Felis catus*	2013	Rio Grande do Sul	Erechim	783 meters	Negative	hII (MF175655)	–	hA (MF175680)
5556	Male	*Canis familiaris*	2014	Rio Grande do Sul	Canela	837 meters	Negative	hIII (MF175656)	h8 (MF175745)	hA (MF175680)
6897A	Female	*Canis familiaris*	2015	Rio Grande do Sul	Igrejinha	18 meters	Negative	hI (MF175654)	–	hA (MF175680)
6897B	Female	*Canis familiaris*	2015	Rio Grande do Sul	Igrejinha	18 meters	Negative	hII (MF175655)	–	hA (MF175680)
6897D	Female	*Canis familiaris*	2015	Rio Grande do Sul	Igrejinha	18 meters	Negative	hV (MF175658)	–	hK (MF175690)
6897E	Female	*Canis familiaris*	2015	Rio Grande do Sul	Igrejinha	18 meters	Negative	hII (MF175655)	–	hA (MF175680)
6897F	Female	*Canis familiaris*	2015	Rio Grande do Sul	Igrejinha	18 meters	Negative	hIV (MF175657)	h9 (MF175746)	hA (MF175680)
6897G	Male	*Canis familiaris*	2015	Rio Grande do Sul	Igrejinha	18 meters	Negative	–	–	hK (MF175690)

COII, cytochrome oxidase subunit II.

Table S4. Sample data, haplotype and associated GenBank accession numbers for *Amblyomma aureolatum* 12S rDNA, cytochrome oxidase subunit II (COII), D-loop and rickettsia sequences analyzed from Brazil (continuation).

Sample number	Tick sex	Host	Year collection	State	Localities	Altitude	Presence of rickettsia (Amplified gene) (GenBank accession number)	12S rDNA Haplotype (GenBank accession number)	COII Haplotype (GenBank accession numbers)	D-loop Haplotypes (GenBank accession numbers)
6897H	Male	*Canis familiaris*	2015	Rio Grande do Sul	Igrejinha	18 meters	Negative	hI (MF175654)	h17 (MT578029)	hA (MF175680)
6897I	Male	*Canis familiaris*	2015	Rio Grande do Sul	Igrejinha	18 meters	Negative	–	–	hK (MF175690)
6897K	Male	*Canis familiaris*	2015	Rio Grande do Sul	Igrejinha	18 meters	Negative	hI (MF175654)	h14 (MT578026)	hA (MF175680)
6897L	Male	*Canis familiaris*	2015	Rio Grande do Sul	Igrejinha	18 meters	Negative	–	h10 (MF175747)	hK (MF175690)
6897M	Male	*Canis familiaris*	2015	Rio Grande do Sul	Igrejinha	18 meters	Negative	hII (MF175655)	h14 (MT578026)	hA (MF175680)
6897N	Male	*Canis familiaris*	2015	Rio Grande do Sul	Igrejinha	18 meters	Negative	–	–	hK (MF175690)
6897O	Male	*Canis familiaris*	2015	Rio Grande do Sul	Igrejinha	18 meters	Negative	–	–	hK (MF175690)
6897P	Male	*Canis familiaris*	2015	Rio Grande do Sul	Igrejinha	18 meters	Negative	–	–	hA (MF175680)
6897Q	Male	*Canis familiaris*	2015	Rio Grande do Sul	Igrejinha	18 meters	Negative	hV (MF175658)	–	hK (MF175690)
6898B	Male	*Canis familiaris*	2015	Rio Grande do Sul	Igrejinha	18 meters	Negative	hII (MF175655)	h15 (MT578027)	hA (MF175680)
6899	Female	*Canis familiaris*	2015	Rio Grande do Sul	Igrejinha	18 meters	Negative	–	–	hA (MF175680)
8123	Female	*Homo sapiens*	2014	Rio Grande do Sul	Palmares do Sul	10 meters	Negative	–	–	hL (MF175691)
8127	Male	*Homo sapiens*	2015	Rio Grande do Sul	Veranópolis	705 meters	Negative	hI (MF175654)	h16 (MT578028)	hA (MF175680)
4201B	Male	*Canis familiaris*	2012	Rio de Janeiro	Itatiaia	1,648 meters	Negative	–	h15 (MT578027)	hB (MF175681)
4928	Female	*Canis familiaris*	2013	Rio de Janeiro	Petrópolis	838 meters	Negative	hII (MF175655)	–	hA (MF175680)
4929	Female	*Canis familiaris*	2013	Rio de Janeiro	Petrópolis	838 meters	Negative	–	–	hA (MF175680)
4930	Female	*Canis familiaris*	2013	Rio de Janeiro	Petrópolis	838 meters	Negative	hII (MF175655)	–	–
4931	Female	*Canis familiaris*	2013	Rio de Janeiro	Petrópolis	838 meters	Negative	–	–	hE (MF175684)
4933	Female	*Canis familiaris*	2013	Rio de Janeiro	Petrópolis	838 meters	Negative	hII (MF175655)	–	hA (MF175680)
4936B	Female	Environment	2013	Rio de Janeiro	Petrópolis	838 meters	Negative	–	–	hA (MF175680)
4936C	Male	Environment	2013	Rio de Janeiro	Petrópolis	838 meters	Negative	–	h5 (MF175742)	hA (MF175680)
4936D	Male	Environment	2013	Rio de Janeiro	Petrópolis	838 meters	Negative	–	h5 (MF175742)	hA (MF175680)
5258	Female	*Canis familiaris*	2013	Rio de Janeiro	Petrópolis	838 meters	Negative	hII (MF175655)	–	hA (MF175680)
5773A	Male	*Canis familiaris*	2014	Rio de Janeiro	Petrópolis	838 meters	Negative	–	h15 (MT578027)	hA (MF175680)
5773B	Female	*Canis familiaris*	2014	Rio de Janeiro	Petrópolis	838 meters	Negative	–	–	hB (MF175681)
6234	Male	*Felis catus*	2014	Rio de Janeiro	Petrópolis	838 meters	Negative	–	–	hA (MF175680)
6235	Female	*Felis catus*	2014	Rio de Janeiro	Petrópolis	838 meters	Negative	–	–	hA (MF175680)
6236	Female	*Canis familiaris*	2014	Rio de Janeiro	Petrópolis	838 meters	Negative	–	–	hF (MF175685)
6237	Male	*Felis catus*	2014	Rio de Janeiro	Petrópolis	838 meters	Negative	–	–	hB (MF175681)
6239	Female	*Felis catus*	2014	Rio de Janeiro	Petrópolis	838 meters	Negative	–	–	hA (MF175680)

COII, cytochrome oxidase subunit II.

Table S4. Sample data, haplotype and associated GenBank accession numbers for *Amblyomma aureolatum* 12S rDNA, cytochrome oxidase subunit II (COII), D-loop and rickettsia sequences analyzed from Brazil (continuation).

Sample number	Tick sex	Host	Year collection	State	Localities	Altitude	Presence of rickettsia (Amplified gene) (GenBank accession number)	12S rDNA Haplotype (GenBank accession number)	COII Haplotype (GenBank accession numbers)	D-loop Haplotypes (GenBank accession numbers)
6249A	Male	*Canis familiaris*	2015	São Paulo	Santo André	760 meters	Negative	hI (MF175654)	h14 (MT578026)	hI (MF175688)
6249B	Male	*Canis familiaris*	2015	São Paulo	Santo André	760 meters	Negative	hI (MF175654)	–	hA (MF175680)
6249B1	Female	*Canis familiaris*	2015	São Paulo	Santo André	760 meters	Negative	hI (MF175654)	–	hA (MF175680)
6249C	Male	*Canis familiaris*	2015	São Paulo	Santo André	760 meters	Negative	–	h15 (MT578027)	hA (MF175680)
6249C1	Female	*Canis familiaris*	2015	São Paulo	Santo André	760 meters	Negative	–		hA (MF175680)
6249D	Male	*Canis familiaris*	2015	São Paulo	Santo André	760 meters	Negative	hII (MF175655)	h6 (MF175743)	hA (MF175680)
6249E	Male	*Canis familiaris*	2015	São Paulo	Santo André	760 meters	Negative	hI (MF175654)	h15 (MT578027)	hH (MF175687)
6249E1	Female	*Canis familiaris*	2015	São Paulo	Santo André	760 meters	Negative	–		hA (MF175680)
6249F	Male	*Canis familiaris*	2015	São Paulo	Santo André	760 meters	Negative	hI (MF175654)	h15 (MT578027)	hI (MF175688)
6249F1	Female	*Canis familiaris*	2015	São Paulo	Santo André	760 meters	Negative	hI (MF175654)	–	–
6249G	Male	*Canis familiaris*	2015	São Paulo	Santo André	760 meters	Negative	hI (MF175654)	h6 (MF175743)	hA (MF175680)
6249H	Male	*Canis familiaris*	2015	São Paulo	Santo André	760 meters	*Rickettsia bellii* (*glt*A, *htr*A) (MF175769/ MF175786)	hII (MF175655)	h15 (MT578027)	hA (MF175680)
6249I	Male	*Canis familiaris*	2015	São Paulo	Santo André	760 meters	Negative	hII (MF175655)	h15 (MT578027)	hA (MF175680)
6249I1	Female	*Canis familiaris*	2015	São Paulo	Santo André	760 meters	*Rickettsia bellii* (*glt*A) (MF175772)	hI (MF175654)	–	hA (MF175680)
6249J	Male	*Canis familiaris*	2015	São Paulo	Santo André	760 meters	Negative	–	–	hA (MF175680)
6249J1	Female	*Canis familiaris*	2015	São Paulo	Santo André	760 meters	Negative	hII (MF175655)	–	–
6249K	Female	*Canis familiaris*	2015	São Paulo	Santo André	760 meters	*Rickettsia bellii* (*glt*A) (MF175770)	hII (MF175655)	h15 (MT578027)	hB (MF175681)
6249K1	Female	*Canis familiaris*	2015	São Paulo	Santo André	760 meters	Negative	hII (MF175655)	–	hG (MF175686)
6249L	Female	*Canis familiaris*	2015	São Paulo	Santo André	760 meters	Negative	hI (MF175654)	h2 (MF175739)	hA (MF175680)
6249L1	Female	*Canis familiaris*	2015	São Paulo	Santo André	760 meters	Negative	hI (MF175654)	–	hA (MF175680)
6249M	Female	*Canis familiaris*	2015	São Paulo	Santo André	760 meters	Negative	hI (MF175654)	–	–
6249M1	Female	*Canis familiaris*	2015	São Paulo	Santo André	760 meters	Negative	hII (MF175655)	–	–
6249N	Female	*Canis familiaris*	2015	São Paulo	Santo André	760 meters	Negative	hI (MF175654)	–	–
6249P	Female	*Canis familiaris*	2015	São Paulo	Santo André	760 meters	Negative	hI (MF175654)	h15 (MT578027)	hA (MF175680)
6249P1	Female	*Canis familiaris*	2015	São Paulo	Santo André	760 meters	Negative	hI (MF175654)	–	hA (MF175680)
6249Q	Female	*Canis familiaris*	2015	São Paulo	Santo André	760 meters	Negative	hI (MF175654)	h7 (MF175744)	hJ (MF175689)
6249Q1	Female	*Canis familiaris*	2015	São Paulo	Santo André	760 meters	Negative	hII (MF175655)	–	hA (MF175680)
6249R	Female	*Canis familiaris*	2015	São Paulo	Santo André	760 meters	*Rickettsia bellii* (*glt*A, *htr*A) (MF175771/ MF175787)	hI (MF175654)	h14 (MT578026)	hA (MF175680)
6249R1	Female	*Canis familiaris*	2015	São Paulo	Santo André	760 meters	Negative	hI (MF175654)	–	–
6249S	Female	*Canis familiaris*	2015	São Paulo	Santo André	760 meters	Negative	hI (MF175654)	–	hA (MF175680)
6249T	Female	*Canis familiaris*	2015	São Paulo	Santo André	760 meters	Negative	hI (MF175654)	h15 (MT578027)	hA (MF175680)
6249T1	Female	*Canis familiaris*	2015	São Paulo	Santo André	760 meters	Negative	hI (MF175654)	–	hA (MF175680)
6249U1	Female	*Canis familiaris*	2015	São Paulo	Santo André	760 meters	Negative	hI (MF175654)	–	hA (MF175680)
6249V	Female	*Canis familiaris*	2015	São Paulo	Santo André	760 meters	Negative	–	h15 (MT578027)	–

COII, cytochrome oxidase subunit II.
