# Supplementary material for: Amblyomma aureolatum Genetic Diversity and Population Dynamics Are Not Related to Spotted Fever Epidemiological Scenarios in Brazil
Source: Pathogens. 2021 Sep 6;10(9):1146. doi: 10.3390/pathogens10091146 (PMC8469259; doi:10.3390/pathogens10091146)
Supplement: Supplementary file 1 [file pathogens-10-01146-s001.zip › Table S5.pdf]

Table S5. Collection data for *Amblyomma aureolatum* samples analyzed from Brazil.

State	Year collection	Biome	Localities	Host	Tick sex
Paraná	2015	Atlantic Rainforest	Irati	*Homo sapiens*	1 female
	2016	Atlantic Rainforest	União da Vitória	*Canis familiaris*	2 females
		Atlantic Rainforest	Paula Freitas	*Canis familiaris*	1 female
		Atlantic Rainforest	Antônio Olinto	*Canis familiaris*	1 female
		Atlantic Rainforest	General Carneiro	Environment	1 female
				Canis familiaris	1 female
	2017	Atlantic Rainforest	Tijucas do Sul	*Homo sapiens*	1 male
				Canis familiaris	6 males
		Atlantic Rainforest	Paulo Frontin	*Homo sapiens*	1 female
		Atlantic Rainforest	General Carneiro	Environment	1 female
				Felis catus	1 male
		Atlantic Rainforest	Lapa	*Homo sapiens*	1 female
		Atlantic Rainforest	Araucária	*Canis familiaris*	1 male
		Atlantic Rainforest	São Mateus do Sul	Environment	2 females
				Canis familiaris	1 female, 1 male
		Atlantic Rainforest	Pinhais	*Canis familiaris*	1 male
		Atlantic Rainforest	Bituruna	*Felis catus*	2 females
		Atlantic Rainforest	General Carneiro	*Canis familiaris*	1 female, 1 male
		Atlantic Rainforest	União da Vitória	*Canis familiaris*	1 female, 1 male
Santa Catarina	2013	Atlantic Rainforest	Lages	*Canis familiaris*	3 males
		Atlantic Rainforest	Joinville	*Nasua nasua*	1 male
		Atlantic Rainforest	Jacinto Machado	*Canis familiaris*	2 females, 3 males
	2014	Atlantic Rainforest	Lages	*Canis familiaris*	2 females, 1 male
		Atlantic Rainforest	Balneário Piçarras	*Canis familiaris*	1 male
		Atlantic Rainforest	Florianópolis	*Homo sapiens*	1 male
		Atlantic Rainforest	Joinville	*Canis familiaris*	2 females
	2015	Atlantic Rainforest	Jaraguá do Sul	*Canis familiaris*	1 male
		Atlantic Rainforest	Tijucas	*Canis familiaris*	1 male
	2017	Atlantic Rainforest	Guabiruba	*Canis familiaris*	1 male
		Atlantic Rainforest	Brusque	*Homo sapiens*	1 female, 1 male
Rio Grande do Sul	2012	Pampa	Viamão	*Canis familiaris*	1 female
	2013	Atlantic Rainforest	São Francisco de Paula	*Canis familiaris*	1 female, 4 males
		Atlantic Rainforest	Erechim	*Felis catus*	1 male
	2014	Atlantic Rainforest	Canela	*Canis familiaris*	1 male
		Pampa	Palmares do Sul	*Homo sapiens*	1 female
	2015	Atlantic Rainforest	Igrejinha	*Canis familiaris*	6 females, 11 males
		Atlantic Rainforest	Veranópolis	*Homo sapiens*	1 male
Rio de Janeiro	2012	Atlantic Rainforest	Itatiaia	*Canis familiaris*	1 male
	2013	Atlantic Rainforest	Petrópolis	*Canis familiaris*	6 females
				Environment	1 female, 2 males
	2014	Atlantic Rainforest	Petrópolis	*Canis familiaris*	2 females, 1 male
				Felis catus	2 females, 2 males
São Paulo	2015	Atlantic Rainforest	Santo André	*Canis familiaris*	24 females, 9 males
TOTAL					68 females, 60 males<br>128 specimens
